# Facile synthesis and eco-friendly analytical methods for concurrent estimation of selected pharmaceutical drugs in their solutions: application to quality by design, lean six sigma, and stability studies

**DOI:** 10.1186/s13065-023-01028-8

**Published:** 2023-10-10

**Authors:** Nada S. Al-Kadhi, Mahmoud A. Mohamed, Hoda A. Ahmed, Hossam F. Nassar

**Affiliations:** 1 Department of Chemistry, College of Science, Princess Nourah Bint Abdulrahman University, P.O. Box 84428, Riyadh, 11671 Saudi Arabia; 2 Hikma Pharmaceutical Company, Beni-Suef, Egypt; 3 Department of Chemistry, Faculty of Science, Cairo University, Cairo, 12613 Egypt; 4 Environmental Science and Industrial Development Department, Faculty of Post Graduate Studies for Advanced Sciences, Beni-Suef University, Beni‑Suef, Egypt

**Keywords:** Pharmaceutical drugs, Ceftazidime, Pyridine, Box-Behnken design, Response surface methodology, Six sigma methodology, Spectrophotometric methods, RP- UPLC, Accelerated stability study

## Abstract

Economical, highly robust, selective, precise, and eco-friendly RP-UPLC and spectrophotometric methods were developed and validated for the concurrent estimation of selected pharmaceutical drugs represented in ceftazidime (CFZ) and pyridine (PYD) in their solutions using Agilent Zorbax SB-C18 RRHD (50 × 2.1 mm, 1.8 μm) column at flow rate 0.3 mL/min with wavelength 254 nm. Box-Behnken design (BBD) established Response surface methodology (RSM) to achieve the optimum chromatographic condition with minimal trials conducted. Three independent variables specifically acetonitrile ratio 60–70%, pH 3–7, and temperature 25–35 °C were implemented to evaluate the influences of these variables on the responses as resolution and retention time. Desirability and overlay plots were carried out to adjust the optimal condition that achieved the shortest retention time of less than 2 min and desired resolution of more than 1.5 using a mobile phase consisting of acetonitrile: purified water (70:30, v/v) at pH 5.0 adjusted by 0.1% orthophosphoric acid with the column oven temperature 30 °C and column void volume 0.46 mL. Mean centering of ratio spectra (MCR) and ratio subtraction (RS) methods were effectively applied to resolve drugs' spectral superposition at 220 nm, 255.4 nm, 260.3 nm, and 254.6 nm for CFZ and PYD, respectively. Linearity range was accomplished for UPLC, MCR, and RS methods over the concentration range of 2–100, 1–50,3–30 and 5–30 µg/mL for CFZ and PYD, respectively with correlation coefficient > 0.999 and good recovery results within 98–102%. Six Sigma methodology was achieved using the process capability index (Cpk) to compare the suggested and USP methods showing that both are highly capable with Cpk > 1.33. The proposed method was successfully validated depending on ICH guidelines and ANOVA results and applied for the accelerated stability study.

**Graphical Abstract:**

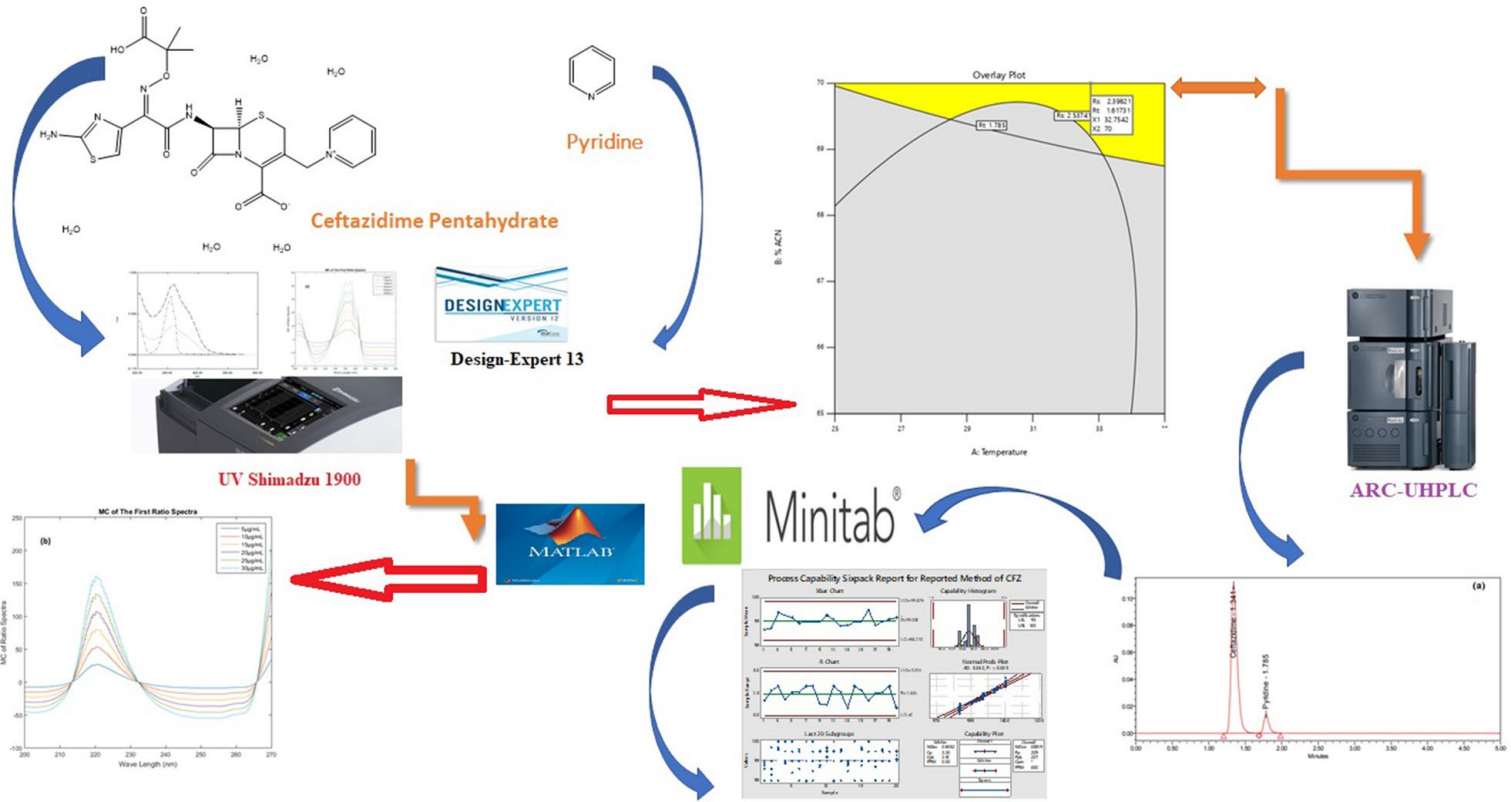

## Introduction

**Fig. 1:**
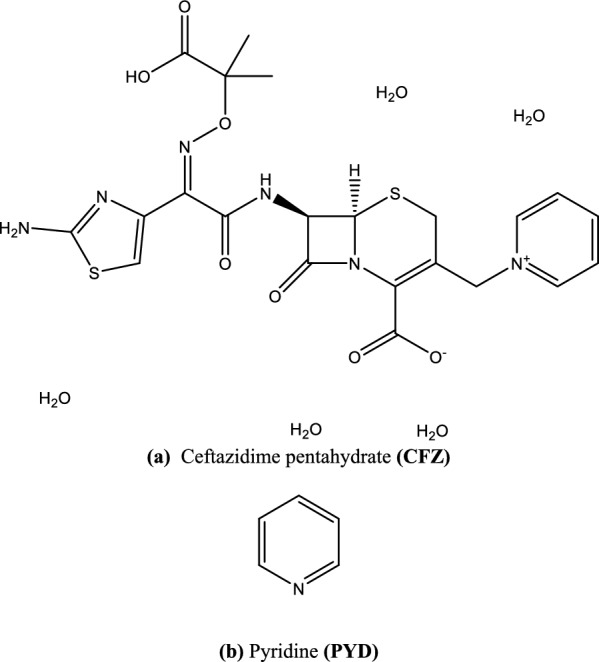
Chemical structures of **a** ceftazidime, and **b** Pyridine

Cephalosporin antibiotic products have been widely detected in the aquatic environments. However, their environmental interactions are poorly understood. Ceftazidime (CFZ) pentahydrate is a semi-synthetic substance developed through a fermentation process that is like a third-generation cephalosporin but has better effectiveness against Gram-negative bacteria. Ceftazidime's activity against Pseudomonas species distinguishes it from other cephalosporins. Ceftazidime is given intravenously (IV) or intramuscularly (IM), and its appearance is described as a white or nearly white, crystalline powder. It has a unique chemical feature for solubility, as it is partially soluble in water and methanol but generally insoluble in acetone and ethanol (96 percent). It is soluble in alkaline and acidic solutions, Fig. 1a [1]. Pyridine (PYD) is also known as azabenzene in organic chemistry. It has the chemical formula C_5_H_5_N and a molecular weight of 79.10. PYD is a colorless, transparent liquid, hygroscopic, and highly soluble in water and ethanol (96 percent). It is also used in manufacturing pharmaceutical drugs, vitamins, and flavors with low concentrations. It is dangerous if breathed, digested, or absorbed via the skin. It is predominantly recognized to decrease spermatogenesis and is carcinogenic, Fig. 1b [1].

The future progress in liquid chromatography is UPLC which increases sensitivity, efficiency, and accuracy. UPLC system uniquely offers amelioration in the detection and separation and decreases the run time tenfold compared to HPLC methods [2]. Response surface methodology is a collection of sophisticated design of experiments (DOE) methodologies. RSM supplies a unique advantage over traditional. RSM can assist in optimizing responses and better understanding the interaction between independent variables through quadratic terms in the polynomial equation. RSM can efficiently reduce the analysis cost by minimizing the number of experimental trials [3–10]. In the recent past, before the implementation of Six Sigma, almost pharmaceutical factories were suffering from high scrap and reprocessing without prior detection of an error, so the applications of the six-sigma methodology have become imperious in pharmaceutical industries to avoid wasting time, removing defects, and increase capability index in the process [11–13]. The stability study aims to offer data concerning how the performance of the pharmaceutical product varies gradually due to environmental variables such as light, humidity, and temperature, as well as to determine the shelf life of the drug product [14]. Using green ideas in analytical chemistry without changing or affecting the merit numbers is hard. As most chromatographic procedures need toxic solvents, eliminating their usage is crucial to greening an analytical method. Nevertheless, there is a trend toward employing "green solvents," which raises additional concerns. We must come up with green ways to identify different environmental toxins because there is a growing need around the world to stop using toxic chemicals and cut down on releases that are bad for the environment. To make analytical procedures safe, dangerous solvents should be swapped out for safer ones, or waste should be eliminated [15–20]. BBD is a novel method for enhancing the precision of experimental outcomes. The method development and validation of the HPLC analytical technique used BBD-optimized chromatographic conditions. These conditions are good for routine analysis. It was common for HPLC procedures to keep changing one chromatographic parameter until the peak resolution was good enough. If more than one parameter is analyzed, more trials are required. Nevertheless, this is manageable using the BBD technique [21].

British Pharmacopoeia (BP) and United States Pharmacopeia (USP) mentioned a different HPLC method for the quantification of CFZ and PYD, respectively [1, 22]. Various HPLC-based analytical techniques for estimating CFZ and PYD alone or combined with other drugs have been described. [23–30], UPLC [31–38], LC–MS [39–45], and Spectrophotometric methods [46–51]. Several analytical HPLC method for concurrent estimation of both pharmaceutical drugs in eye drops formulation [52–55] and intravenous (IV), intramuscular (IM) [56–59].

The uniqueness of this study lies in the development of a robust UPLC method for concurrent estimation of a binary mixture in a single run using BBD, which requires fewer trials and is more efficient than the traditional "one factor at a time and green solvents, which have many benefits for cost savings, reduced waste, removing expensive treatment, toxic waste disposal, and sustaining health and safety. Moreover, RS and MCR methods were performed to solve the overlapping in the binary mixture without a sophisticated program and accomplishment of six sigma to evaluate the proposed method and the referenced USP one showing that CpK > 1.33 for both. To our endeavor, no UPLC, RS, and MCR methods were reported to simultaneously determine of CFZ and PYD in their powder for the injection dosage form. So, the essential objective of the current work is to establish novel, robust, and rapid RP-UPLC, RS, and MCR methods for the concurrent quantification of both drugs and the application of six sigma and accelerated stability studies.

## Experimental

### Instrument

The ACQUITY Arc System is upgraded by Waters (Massachusetts, USA) to achieve the linking and easily switch between HPLC and UHPLC. Arc UHPLC is supplied with a UV detector, column manager with a temperature range (4–90 °C) to accomplish the robustness of method development, autosampler needle which decreases carryover by constantly washing the needle during injections and Empower 3 software for processing and reporting.

The UV-1900i was devised by (Shimadzu, Japan) to cater to high accuracy and usability requirements with a wavelength range from 190 to 1100 nm and provided Lab Solutions UV–Vis’s software used for measurements and data analysis.

The stability cabinet (Weiss Technik, Reiskirchen, Germany) was annually verified for keeping the drug products and equipped with SIMPATI software for data monitoring and trending of temperature and relative humidity, also creation of a stability program was managed using the SAP QM module.

pH meter (Mettler-Toledo, Columbia, USA) was employed for measuring the pH in the solution and calibrated before use by three consecutive buffers (4, 7, 10) with an acceptable limit of 90–105%.

Design-Expert software version 13 was used for approaching response surface methodology and quality by design, MATLAB 2015a was established for handling the mean centering method, Six Sigma, and the process capability index was evaluated by using Minitab 2018 software.

## Materials and reagent

CFZ USP reference standard (Lot No# R08540) was purchased from the USP store with a potency of 98.9% as an anhydrous base. Kefadim 500 mg (B. No #183,066) and Kefadim 1.0 g (B. No # 183,092) were provided by hikma pharmaceutical company (Beni-Suef, Egypt). Pyridine, orthophosphoric analytical grade, and acetonitrile HPLC grade were procured from (Scharlau, Spain). syringe filters (PTFE) 0.45 ϻm and membrane filter (GHP) 0.2 μm were procured from Noor Scientific & Trade, the authorized agent for Merck-Millipore in Egypt.

## Procedures

### Solvent preparation

#### For UPLC method

Acetonitrile: Acidic water adjusted at pH 5.0 (10:90).

#### For UV methods

0.1 N HCl

### Preparation of working standard solutions (Laboratory prepared mixture)

Carefully weigh 58 mg of CFZ pentahydrate reference standard is equivalent to 50 mg CFZ base and 7 mg of PYD in a 200 mL volumetric flask, add 75 mL from solvent and sonicate for 5 min and complete to mark, then dilute 10 mL from the stock solution and transfer to 100 mL volumetric flask and filter through 0.45 µm PTFE syringe filter to obtain the final concentration of 25 µg/mL from CFZ and 3.5 µg/mL PYD, respectively.

### Test preparation

Weigh and disperse ten filled vials and determine the average content per vial. Transfer accurately weighed quantity 290 mg containing the equivalent to 250 mg CFZ and 35 PYD, respectively in 500 mL volumetric flask, dissolve on sonication for 5 min. Transfer 5 mL of this solution into a 100 mL volumetric flask. Discard the first 4 mL, then filter through a PTFE filter of 0.45-µm.

## Procedure

### Chromatographic conditions

The isocratic system was established on Arc UPLC for the separation of the UPLC method for concurrent estimation of CFZ and PYD using RRHD Zorbax SB-C18 column (50 × 2.1 mm, 1.8 μm) and a mobile phase consisting of acetonitrile: purified water (70:30, v/v) at pH 5.0 adjusted by 0.1% orthophosphoric acid with flow rate 0.3 mL/min, column oven temperature 30 °C, and injection volume 0.3 µL at UV detection 254 nm. The mobile phase was filtered through a 0.2 µm membrane filter (GHP) and degassed for 10 min on the ultrasonic bath. All solvent lines and seal wash were primed by gradient wash with the following sequence (i) 15 min with mobile phase, (ii) 40 min with (20% organic: 80% H_2_O), 30 min with (70% organic: 30% H_2_O) to avoid air bubbles and buffer precipitations in the instrument tubing.

### Calibration curves construction

A series dilution of 10 mL volumetric flasks was taken from the standard stock solution to obtain the final concentration range of 2–100 and 1–50 µg/mL for CFZ and PYD in the UPLC method and 3–30, 5–30 µg/mL for CFZ and PYD, respectively in RS and MCR methods. The sequence was started by six system suitability injections, two injections of standard solution (1, 2), two injections of Test solution (1, 2, 3), and two injections of standard check #01. Data processing and sign-off reports were performed on Empower 3 software for the sample set, the calibration curves were established versus the relevant concentrations and the regression equations were calculated for UPLC, RS, and MCR techniques.

### Design of experiment for optimizing RP-UPLC method

Essential parameters affected directly by chromatographic separation were evaluated in the initial screening analysis. Three variables at three levels of BBD with RSM were implemented for optimizing chromatographic conditions with good resolution and the shortest retention time for both drugs with the least number of experimental runs. Independent factors of pH, the ratio of acetonitrile in the mobile phase, and column oven temperature were selected. The retention time and resolution were used as responses. Experimental 17 runs were displayed in Table 1. The second-order polynomial equation obtained from ANOVA results describes the quadratic models. Further, a 3D response surface was used to indicate the independent variables' interaction. Finally, the desirability function and overlay plots were employed to predict the optimum final condition.

**Table 1.** Factors and responses for Box-Behnken experimental design of the optimized method

Std.	Run	Factor 1	Factor 2	Factor 3	Response 1	Response 2
		A: Temperature	B: % ACN	C: pH	Rs	Rt
15	1	30	65	5	3.22	2.514
14	2	30	65	5	3.22	2.514
13	3	30	65	5	3.22	2.514
10	4	30	70	3	2.25	1.785
8	5	35	65	7	1.11	2.714
1	6	25	60	5	3.84	3.416
3	7	25	70	5	2.12	1.81
9	8	30	60	3	4.25	3.318
2	9	35	60	5	1.05	3.205
5	10	25	65	3	4.54	2.874
12	11	30	70	7	1.82	1.824
4	12	35	70	5	1.98	1.802
11	13	30	60	7	0.98	4.281
16	14	30	65	5	3.22	2.514
6	15	35	65	3	3.08	2.415
17	16	30	65	5	3.22	2.514
7	17	25	65	7	0.88	3.198

### Application of six sigma methodology

The main advantage of the process capability index is that companies will understand process behavior to decrease scrap, increase product quality and consistency, and reduce production costs and cost loss due to poor quality. A high process capability index (Cpk) indicates how near a process is to its specified center limit against the process's considerable variation. The low Cpk value indicates that the process needs improvement and can be calculated as follows [60, 61].

$$\text{Cpk}= Min \frac{{\left( {\overline X - LSL} \right)}}{{3\sigma }} or Min \frac{{\left( {USL - \overline X} \right)}}{{3\sigma }}$$

Where; ${\overline{X }}$ is the mean of process, LSL is the lower specification limit, USL is the upper specification limit, and $\sigma$ is the standard deviation of the process.

Cpk always ≤ Cp (Process Capability).

Cpk = Cp when the method is correctly focused.

If a Cpk is negative, it means that the process average has exceeded the specified limit.

If a Cpk equals 0, the processes mean is close to the prescribed limits.

If the Cpk is even less than 1.0, the process does not match the specification limit.

If a Cpk is equal to 1.0 indicates that the process meets the specification limit.

A Cpk of 1.33 [4 sigma] or greater is required to satisfy most clients.

### Ratio subtraction method

The primary benefit of the ratio subtracting method is the ability to perform simple measurements and solve the interfering spectra of a binary mixture. The scanned spectra of both drugs (X) and (Y) were divided by the chosen concentration of 10 μg/mL (Y) as a divisor, and new ratio spectra with constant were obtained. Average absorbance values (1.026) were subtracted in the plateau region (242–260 nm), the resulting spectra were then multiplied by the divisor of 10 g/mL (Y). Lastly, the new zero-order (X) was gained at 260.3 nm. The absorbances of zero-order spectra were calibrated against the appropriate concentrations, and regression equations were constructed at 260.3 nm. Also, (Y) could be obtained by dividing the stored spectra of the combination on the selected concentration 10 μg/mL (X) as a divisor. Then, constant values (1.051) were subtracted in the plateau region (275–320 nm), and the gained spectra were multiplied by the divisor 10 μg/mL (X), resulting in new original spectra of (Y) at 254.6 nm [62].

### Mean centering of ratio spectra method

An advanced spectrophotometric method was implemented to overcome the overlapping found between the binary mixture of CFZ and PYD based on mean centering ratio spectra without prior derivative steps [63].

Assume a three-dimensional vector (X) to illustrate the mean centering equation [64].

$${\text{X}}= \left[ {\begin{array}{ccccccccccccccc}3\\2\\1\end{array}} \right]$$

The mean vector of X is

$${\overline{\text{X}}}= \left[ {\begin{array}{ccccccccccccccc}2\\2\\2\end{array}} \right]$$

The vector S's mean centering could be expressed as

$${\rm{MC}}\left( {\rm{S}} \right) = {\rm{X}} - {\bar { \rm X}}\left[ {\begin{array}{ccccccccccccccc}3\\2\\1\end{array}} \right] - \left[ {\begin{array}{ccccccccccccccc}2\\2\\2\end{array}} \right] = \left[ {\begin{array}{ccccccccccccccc}{ + 1}\\0\\{ - 1}\end{array}} \right]$$

It is simple to establish that if a vector v is multiplied by a constant n, the mean center vector is likewise multiplied by n and that if n is added to the vector v, the mean center does not change. The total absorbance of a combination of binary mixture CFZ and PYD without interaction and obeying Beer's low may be calculated as follows:

1 $${\text{Abx}} = \alpha_{CFZ} C_{CFZ} + \alpha_{PYD} C_{PYD}$$

where $\mathrm{Abx}$ is the absorbance vector of the combination, ${C}_{CFZ}$ and ${C}_{PYD}$ are the concentrations of the relevant drugs, and the molar absorptivity of these drugs are ${\alpha }_{CFZ}$ and ${\alpha }_{bPYD}$*.*

1st spectrum ($CFZ$) is obtained by dividing the absorbance vector of the mixtures by ${\alpha }_{PYD}$

2 $$CFZ = \frac{{{\text{Abs}}\left( {{\text{Mix}}} \right)}}{{\alpha_{PYD} }} = \frac{{\alpha_{CFZ} C_{CFZ} }}{{\alpha_{PYD} { }}} + C_{PYD}$$

Zero values of ${\alpha }_{CFZ}$ disregarded for completing the dividing step, the consequence of MCR on a constant is zero. Therefore,

3 $${\text{MC}}\left( {C_{CFZ} } \right) = {\text{MC}}\left( {C_{PYD} } \right) = { }0{ }$$

By operating the MCR on Eq. (2), thus

4 $${\text{MC}}\left( {CFZ} \right) = {\text{ MC}}\left[ {\frac{{\alpha_{CFZ} C_{CFZ} }}{{\alpha_{PYD} { }}}} \right]$$

Equation (4) is the statistical base of binary mixture analysis that declares the estimation of the concentration of each drug without overlapping with the other drug. A calibration curve was constructed individually or in a binary mixture by graphing MC (CZF) against the concentration of CFZ. Calibration curves for PYD were computed in the same manner for CFZ.

## Stability studies

An accelerated stability study was conducted to study degradation pathways and estimate shelf life by using extreme conditions of high temperature and humidity. Intermediate stability must be performed if there is a substantial change such as decreasing assay results in the accelerated stability at any point by more than 5%, and intermediate stability samples should be kept for one year [65].

## Results and discussion

### Preliminary study

**Fig. 2:**
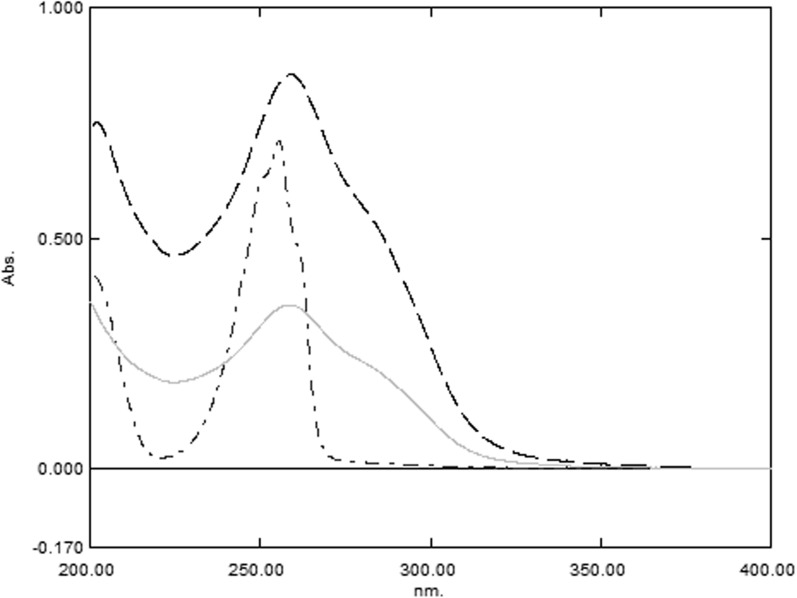
Zero order absorption spectra of 10 µg/mL of CFZ (—), 20 µg/mL of PYD (-----), and 30 (— -) µg/mL of Kefadim 500 mg IV/IM using Solvent as blank

The main objective of the suggested work is to progress a specific and robust UPLC method for concurrent estimation of binary mixture CFZ and PYD in their powder for injection with good resolution and shortest retention time. So, various trials were executed to select the best wavelength and column type and adjust the mobile phase ratio. Scanning wavelength at 200–400 nm for a concentration of 10 µg/mL of CFZ, 20 µg/mL of PYD, and 30 µg/mL of dosage form using 0.1N HCl as blank (Fig. 2), showing that the best wavelength was 254 nm regarding high sensitivity and minimal noise. Varied column types were evaluated including CORTECS Shield C18 (50 mm × 2.1 mm, 1.6 μm), Zorbax SB-C18 RRHD (50 × 2.1 mm, 1.8 μm), and ACE C18 (5 cm × 2.1 mm, 2.0 μm) columns. Preliminary data fixed that the Agilent Zorbax SB-C18 RRHD column (50 × 2.1 mm, 1.8 μm) efficiently separated the examined drugs with the least void volume. Different flow rates ranging from 0.1 to 1 mL/min were tested; the flow rate of 0.3 mL/min demonstrated the quickest elution with good separation. The optimal mobile phase for the concurrent estimation of both drugs was found to be acetonitrile and purified water adjusted with 0.1% orthophosphoric acid. Altering the acetonitrile concentration, pH, and temperature significantly changed the retention time. Column temperature, pH, and the ratio of acetonitrile in the mobile phase were chosen as the crucial variables because they were discovered to have the most effective influence on responses.

### Design of experiments (DoE)

DoE technique with BBD was implemented to adjust the optimum chromatographic conditions with the least number of trials and to study the quadric effect and the interaction between the variables and responses. The factors pH, column temperature, and acetonitrile ratio in the mobile phase were identified, while retention time and resolution were chosen as responses. Linear polynomial equations were investigated for a better grasp of the effects of engagement between independent factors and responses. Listed below is a depiction of the linear polynomial equations that ANOVA generated:

5 $$\begin{aligned} {\text{RS}} & = + 3{\text{.22}} - {0}{\text{.5200}}\;{\text{Temp}} - 0.2438 \% {\text{ACN}} - {1}{\text{.17pH}} \\ &\quad+ 0{.6625}\;{\text{Temp}}* \% {\text{ACN}} + 0{.4225}\;{\text{Temp*pH}} \hfill \\ &\quad +  \;{0}{\text{.7100}}\;\%{\text{ACN*}}\;{\text{pH}}-{0.4475}\;{\text{Temp}}^2 \\ &\quad-0.5250 \% {\text{ACN}}^2 - 0.3700\;{\text{pH}}^2 \hfill \\ \end{aligned}$$

6 $$ \begin{aligned} {\text{Rt}} & = +  2.51 - 0.1453\;{\text{Temp}} - 0.8749\%{\text{ACN}}\\ & \quad  + {\text{0.2031}}\;{\text{pH}}{ + }{0}{\text{.0507Temp*\%ACN}} \hfill \\ & \quad - 0.0063{\text{Temp*pH}} - 0.2310\%{\text{ACN*pH}}+ 0{\text{.0213Temp}}^2 \\ & \quad  + 0.0230\%{\text{ACN}}^2 + 0.2650{\text{pH}}^2 \hfill \\ \end{aligned} $$

**Fig. 3:**
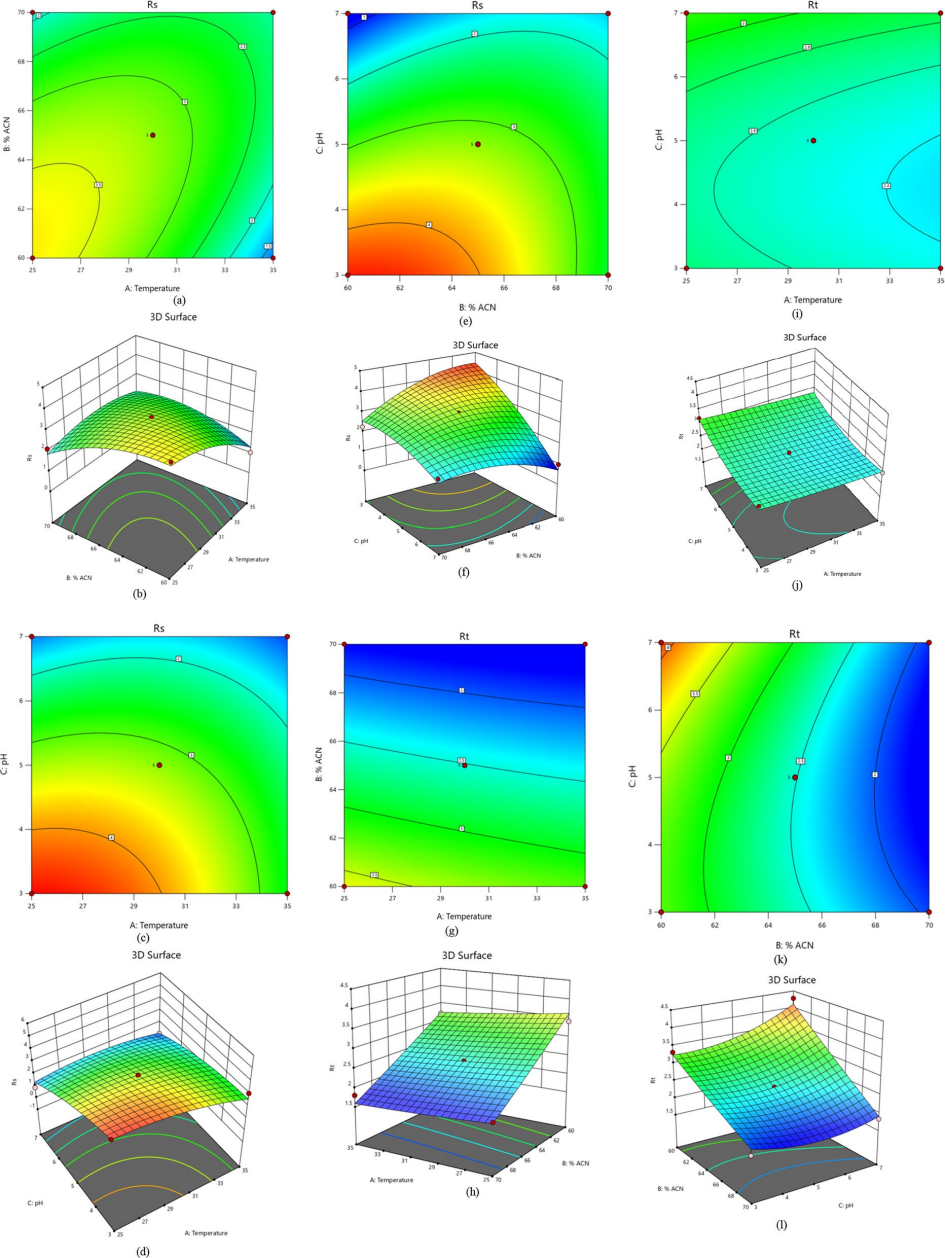
Contour and 3D-response surface plots **a**, **b** the effects of (% ACN) and temperature, **c**, **d** pH and temperature, **e**, **f** pH and (% ACN) on resolution, respectively **g**, **h** the effects of temperature and (% ACN), **i**, **j** pH and temperature, **k**, **l** pH and (% ACN) on retention time

**Fig. 4:**
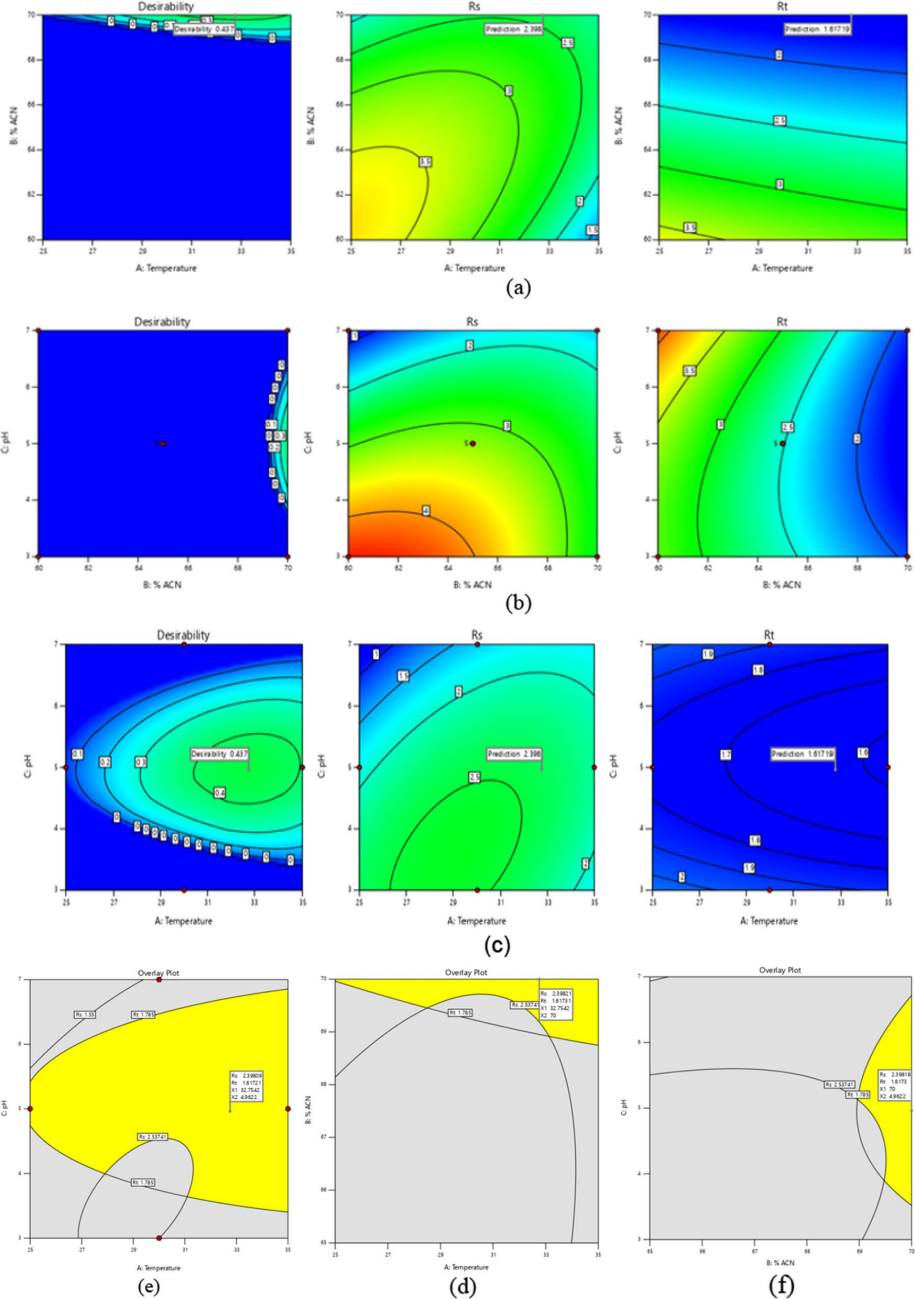
**a**–**c** Desirability and **e**–**f** overlay plots predicted responses

**Fig. 5:**
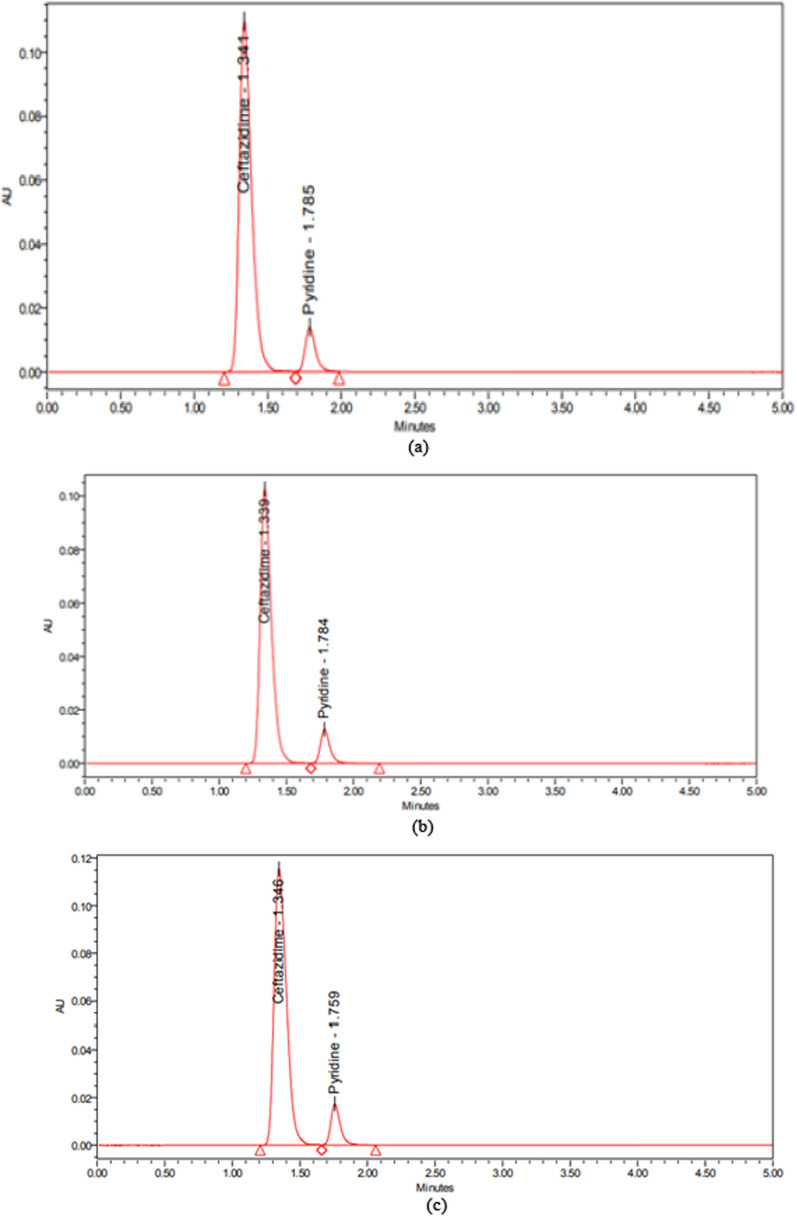
UPLC Chromatograms of **a** standard solution of a mixture of CFZ and PYD, **b** Kefadim 500 mg IV/IM, **c** Kefadim 1000 mg IV/IM

The observed data from Eq. (5) demonstrates that the independent variables with a negative sign negatively influence on the resolution. A positive sign in the interaction terms indicates that the two variables interact positively as shown in (Fig. 3a–f) for Contour and 3D-response surface plots. Equation (6) shows that column temperature and the ratio of acetonitrile have a negative impact on retention time. In contrast, the quadratic effects have a positive effect implying that any slight increase in column temperature and acetonitrile ratio from low to high levels resulted in a decrease in retention time as depicted in (Fig. 3g–l). ANOVA data for resolution and retention time responses displayed in Table 2 shows that the probability P-value < 0.05 means that the model and terms were significant. The R-squared and adjusted values were 0.96, 0.97, 0.91, and 0.94 with a standard deviation less than 0.4 and a lack fit of 0.8440 and 0.2038 for resolution and retention time responses, respectively, indicating that experimental responses were an exemplary appropriate. The numerical optimization function was employed to predict the responses and accomplish the best separation parameters by maximizing desirability to obtain good resolution > 1.5 and retention time less than 2.0 min, as shown in Fig. 4a–c. Overlay plots (Fig. 4d–f) displayed the best variables that led to the desired responses. Parameters were implemented in the laboratory to verify the predicted method. The optimum chromatographic system was acetonitrile: purified water (70:30, v/v) at pH 5.0 adjusted by 0.1% orthophosphoric acid at 30 °C as good resolution, asymmetric peak, and shorter retention time as depicted in Fig. 5.

**Table 2:** ANOVA data for resolution and retention time responses

Source	Sum of Squares	df	Mean Square	F-value	P-value	
Resolution						
Model	20.88	9	2.32	19.24	0.0004	Significant
A-Temperature	2.16	1	2.16	17.94	0.0039	
B-% ACN	0.4753	1	0.4753	3.94	0.0875	
C-pH	10.88	1	10.88	90.25	< 0.0001	
AB	1.76	1	1.76	14.56	0.0066	
AC	0.7140	1	0.7140	5.92	0.0452	
BC	2.02	1	2.02	16.72	0.0046	
A^2^	0.8432	1	0.8432	6.99	0.0332	
B^2^	1.16	1	1.16	9.63	0.0173	
C^2^	0.5764	1	0.5764	4.78	0.0650	
Residual	0.8440	7	0.1206			
Lack of Fit	0.8440	3	0.2813			
Pure Error	0.0000	4	0.0000			
Cor Total	21.72	16				
Retention time						
Model	7.15	9	0.7948	27.30	0.0001	Significant
A-Temperature	0.1688	1	0.1688	5.80	0.0469	
B-% ACN	6.12	1	6.12	210.35	< 0.0001	
C-pH	0.3301	1	0.3301	11.34	0.0120	
AB	0.0103	1	0.0103	0.3539	0.5706	
AC	0.0002	1	0.0002	0.0054	0.9436	
BC	0.2134	1	0.2134	7.33	0.0303	
A^2^	0.0019	1	0.0019	0.0653	0.8056	
B^2^	0.0022	1	0.0022	0.0765	0.7901	
C^2^	0.2957	1	0.2957	10.16	0.0153	
Residual	0.2038	7	0.0291			
Lack of Fit	0.2038	3	0.0679			
Pure Error	0.0000	4	0.0000			
Cor Total	7.36	16

### Ratio subtraction method

**Fig. 6:**
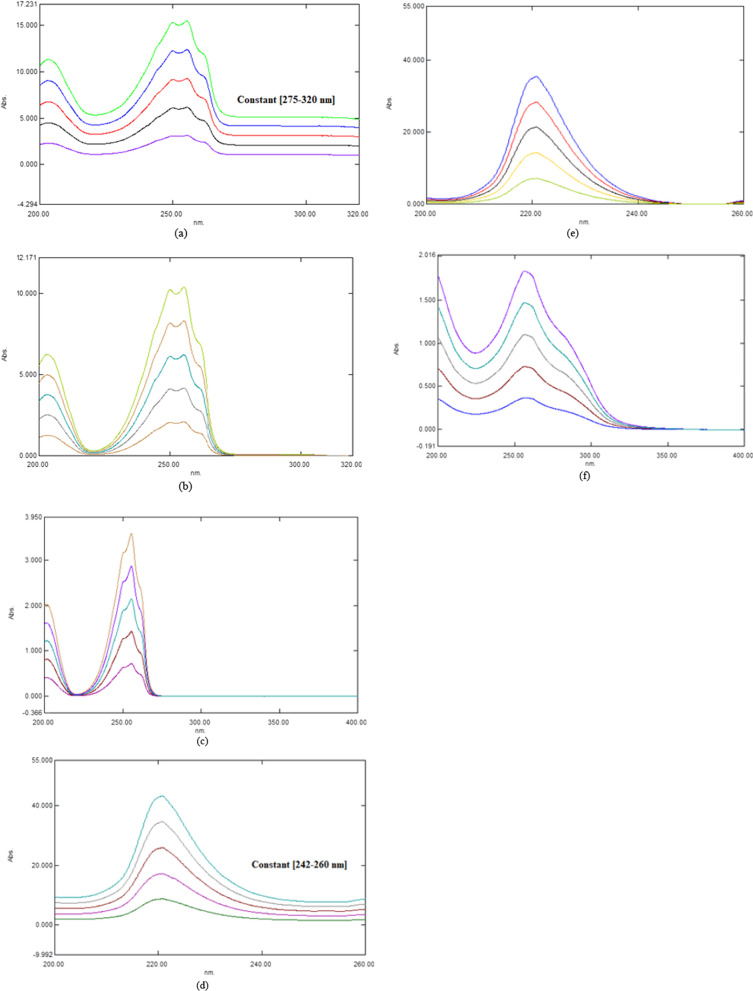
**a**, **d** ratio spectra of CFZ and PYD using 20 µg/mL of CFZ and 5 µg/mL of PYD as divisors, respectively **b**, **e** ratio spectra of CFZ and PYD after subtraction of constant, and **c**, **f** zero order spectra of CFZ and PYD after multiplication by the divisors

The scanned spectra of the laboratory prepared mixture were divided by the chosen concentration of 10 μg/mL for CFZ (divisor). As a result of this, a new ratio spectrum was formed. The obtained average absorbance values (1.026) were subtracted in the plateau area (275–320 nm) as depicted in (Fig. 6a, b), The obtained spectra were therefore multiplied by a divisor. Ultimately, new zero order spectra were obtained for PYD at 254.6 nm as displayed in Fig. 6c. Linear calibration curves were generated by graphing absorbance values for the zero order spectra of PYD at 254.6 nm against the relevant concentrations and then computing the regression equations as displayed in Table 2. Furthermore, the new spectra of CFZ were gained by dividing the prepared mixture of both drugs by the selected concentration of 10 μg/mL of CFZ (divisor), then the average absorbances values (1.151) of the constant were subtracted in the plateau area (242–260 nm) as depicted in (Fig. 6d, e). Lastly, new CFZ spectra were produced at 260.3 nm by multiplying the previous CFZ spectra by the divisor, Fig. 6f. The calibration graph was constructed by graphing absorbance values of zero order against their concentrations and the regressing equation is calculated, Table 3.

**Table 3:** Regression and validation items of the suggested UPLC and UV methods for estimation of CFZ and PYD

Parameter	UPLC		RS		MCR	
	CFZ	PYD	CFZ	PYD	CFZ	PYD
Wavelength	254 nm	254 nm	260.3 nm	254.6 nm	220 nm	255.4 nm
Range (µg/mL)	2–100	1–50	3–30	3–30	5–30	5–30
Slope	12,810	2792.5	0.0826	0.0409	31.947	15.295
Intercept	2402.4	394.3	0.0053	0.0005	1.1071	0.643
Correlation coefficient	0.9999	0.9998	0.9998	0.9997	0.9999	0.9999
Repeatability	0.4	0.7	0.2	0.3	0.1	0.2
LOD^a^ (µg/mL)	0.68	0.29	0.3	0.38	0.2	0.3
LOQ^a^ (µg/mL)	2.08	0.87	1.15	0.91	0.91	0.6

^a^Limit of detection (3.3 × σ /Slope) and limit of quantitation (10 × σ /Slope)

### Mean centering of ratio spectra method

**Fig. 7:**
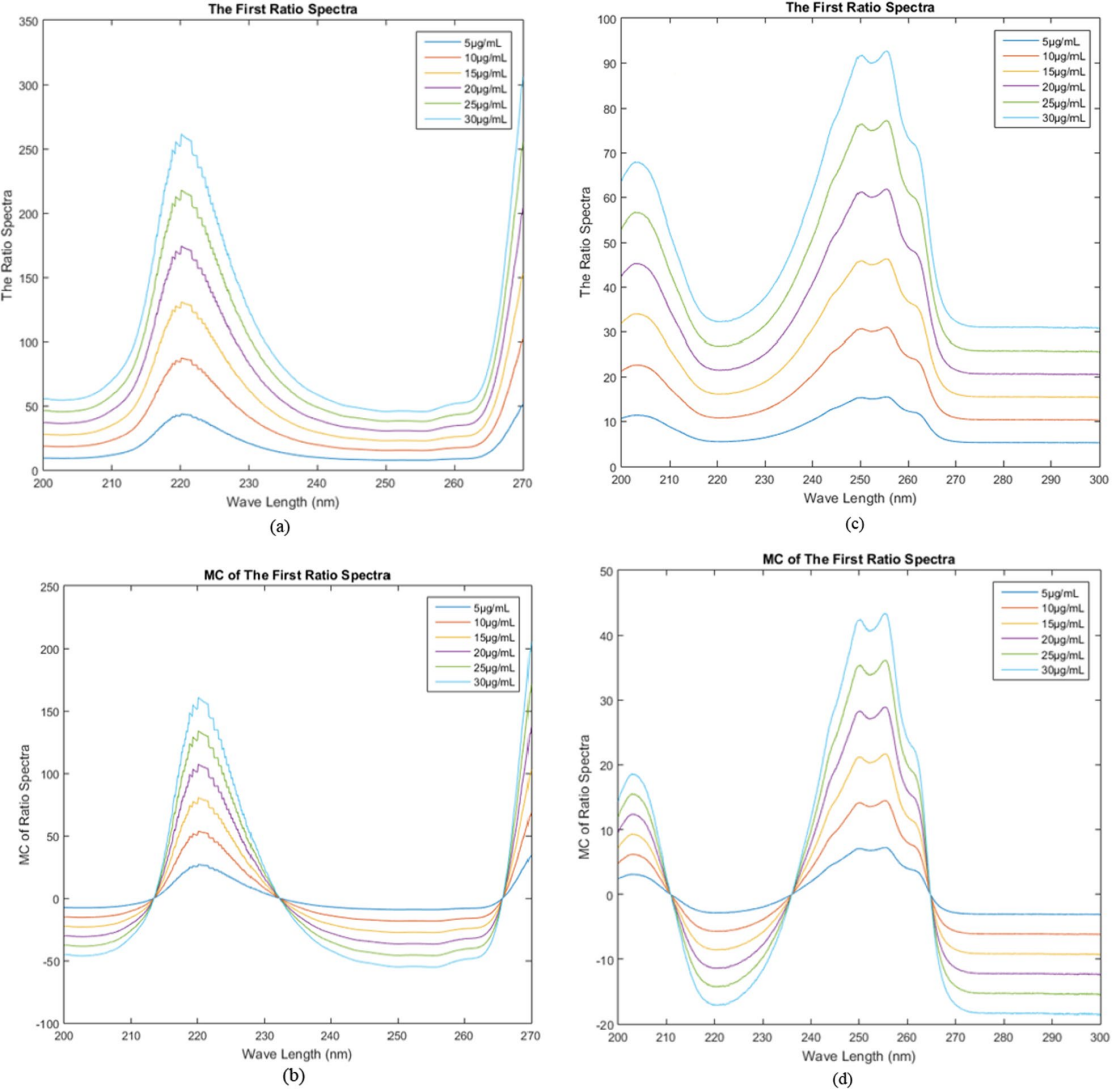
**a**, **b** First ratio spectra of CFZ and PYD, and **c**, **d** Mean centered ratio spectra of CFZ and PYD

The MCR method was employed to separate CFZ and PYD drugs in a combination of a binary mixture in one shot without pre-separation. Several divisor concentrations were examined to enhance the suggested method. The most appropriate concentration of the divisor is 5 µg/mL. The drug spectra were generated in the range 200–270 nm for CFZ and 200–300 nm for PYD, as depicted in Fig. 7a, c. The absorbance values of 5–30 µg/mL of CFZ, PYD, and the laboratory-prepared combination were imported into the MATLAB software. Equations from 1 to 4 were applied to determine MCR for each drug without interfering with the other. As shown in (Fig. 7b, d), CFZ and PYD were estimated at suitable wavelengths 220 and 255.4 nm, respectively. The calibration curves were established by graphing the obtained amplitudes against their respective concentrations, and regression equations were calculated as in Table 3.

### Process capability sixpack

**Fig. 8:**
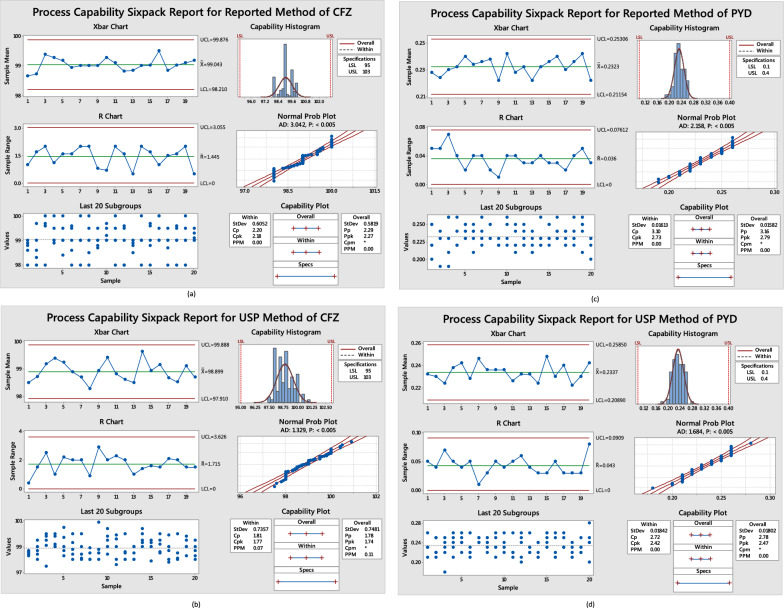
Process capability sixpack for normally distributed assay for comparison between different methods, **a**, **b** (proposed & USP) for CFZ, **c**, **d** (proposed & USP) for PYD using Minitab^®^18

Trend assay results for the proposed and USP methods of CFZ and PYD of 100 batches for Kefadim IV/IM for the last quarter of 2022 were collected and analyzed using Minitab®18.1.0. As shown in Fig. 8, interprets process capability six-pack report generated from quality tools indicates that the process is consistent in both X bar and R charts, with no points outside the control limitations. The last 20 subgroups plot shows that the data are randomly dispensed and uniformly around the processing center. Histogram, average probability, and capability plots show that the process is roughly centered on the target and that the results are within the specified limitations. The Cpk value of CFZ and PYD in USP and proposed methods is greater than 1.33, indicating that both methods are significant and the proposed method more accurate than the USP method as the Cpk values in the proposed method are 2.18, 2.73 (Fig. 8a, c), while in USP methods are 1.77, 2.42 (Fig. 8b, d) for CFZ and PYD, respectively. As shown in Table 4, the values of variance, standard error, and deviation in the proposed method are less than in the USP method.

**Table 4:** Descriptive statistics for the process capability sixpack of the proposed and USP methods

Parameters	CFZ		PYD	
Descriptive statistic	USP method	Proposed method	USP method	Proposed method
Minimum	97.5	98.0	0.18	0.19
Maximum	100.9	100	0.28	0.26
Mean	99.03	99.04	0.24	0.23
Limit	90–105%		NMT 0.4%	
Sum	9889.9	9904.3	23.37	23.23
Count	100	100	100	100
Standard Error	0.075	0.058	0.002	0.002
Median	98.95	99	0.23	0.23
Mode	98.99	99	0.23	0.23
Standard Deviation	0.75	0.58	0.02	0.02
Variance	0.56	0.34	0.0003	0.0003
Kurtosis	− 0.61	− 0.5	− 0.28	− 0.11
Skewness	0.38	0.27	− 0.08	− 0.12
Range	3.4	2	0.10	0.07

### Accelerated stability study

**Fig.9:**
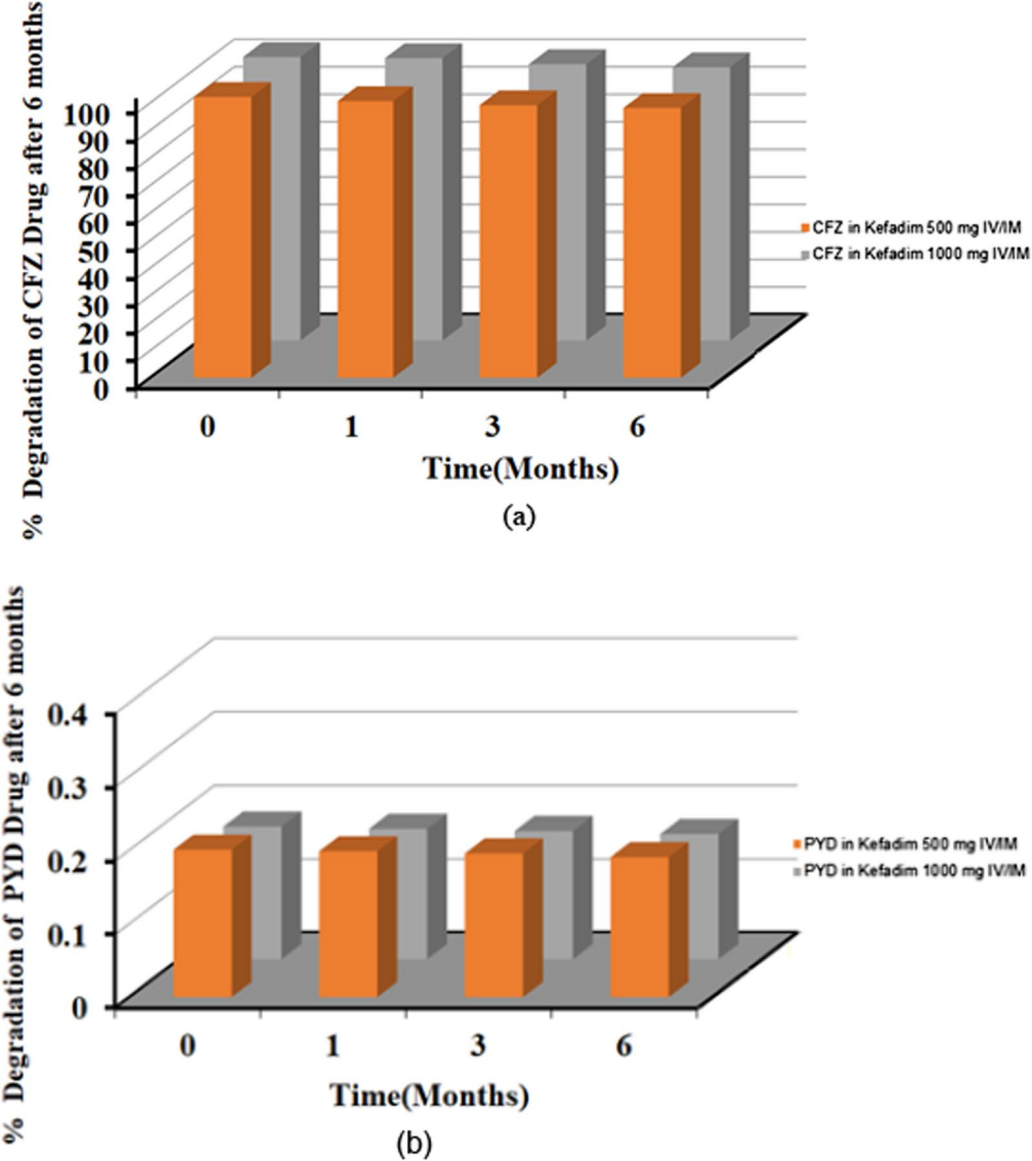
**a** assay results of CFZ in Kefadim 500 mg IV/IM and Kefadim 1000 mg IV/IM Stored for 6 Months at Accelerated conditions, and **b** assay results of PYD in Kefadim 500 mg IV/IM and Kefadim 1000 mg IV/IM Stored for 6 Months under the same conditions

Assay summary results in Table 5 of the accelerated stability study for intervals (0,1, 3, and 6 months) indicate that neither significant change nor degradation by more than 5% of active pharmaceutical ingredient (API) assay from the initial value and the results are capable through the study, Fig. 9a, b.

**Table 5:** Accelerated Stability data for CFZ and PYD in pharmaceutical solution after 6 months

Parameter	Kefadim 500 mg IV/IM (CFZ)	Kefadim 1000 mg IV/IM (CFZ)	Kefadim 500 mg IV/IM (PYD)	Kefadim 1000 mg IV/IM (PYD)	Reference Value
0	102.01	103.00	0.200	0.180	90–110% for CFZ NMT 0.4% for PYD
1	100.50	102.60	0.198	0.177	
3	99.00	100.40	0.195	0.174	
6	98.00	99.30	0.190	0.170	
Average	99.88	101.33	0.195	0.175	
RSD %	1.76	1.75	2.22	2.43

### Method validation

The suggested MCR, UPLC, and RS methods were performed and validated as per ICH recommendation [66].

#### Linearity and range

Linearity test was established for UPLC, RS, and MCR methods over the concentration range (2–100), (1–50), (3–30), and (5–30) µg/mL for CFZ and PYD, respectively. Each concentration was injected in triplicates. The obtained data appeared good linearity results with a correlation coefficient > 0.9996, see Table 3.

#### Limit of detection and quantitation

Detection (LOD) and quantitation limit (LOQ) were executed on a validated Excel sheet using the formula (3.3σ/S) and (10σ/S) for the calculation LOD and LOQ, respectively, where σ refer to the standard deviation of the intercept and S to the slope of the calibration curve. The lower the LOQ and LOD values, the better sensitivity of the proposed methods, as displayed in Table 3.

#### Accuracy and recovery

The accuracy of the proposed UPLC, RS, and MCR methods was established for the studied drugs by preparing three different concentrations (10, 15, and 20 µg/mL) for each drug in triplicate. Good recovery results were obtained and complied with the acceptance criteria (98.0–102.0% with RSD less than 2.0) as shown in Table 6.

**Table 6:** Accuracy and recovery results for CFZ, and PYD by the suggested UPLC and UV methods

CFZ Standard Solution (µg/ mL)	PYD Standard Solution (µg/ mL)	UPLC recovery%		UV recovery%			
				RS		MCR	
		CFZ	PYD	CFZ	PYD	CFZ	PYD
10	10	101.96	101.77	100.72	98.60	99.63	100.41
		101.90	101.85	100.84	98.85	99.94	100.41
		101.84	101.58	101.09	99.09	100.25	99.75
15	15	101.22	101.04	100.92	98.71	99.98	98.75
		101.25	101.06	100.84	98.88	99.77	99.19
		101.33	100.87	100.75	99.37	100.19	98.32
20	20	100.90	99.81	100.71	99.38	99.53	99.56
		100.90	99.91	100.65	99.75	99.69	99.89
		101.24	100.10	100.59	99.63	100.31	99.24
Accuracy (Mean) ± RSD		101.39 ± 0.40	100.89 ± 0.78	100.79 ± 0.15	99.14 ± 0.41	99.92 ± 0.29	99.50 ± 0.71

#### Assay of pharmaceutical formulation

The Assay test solution was evaluated by preparing three samples in duplicate and applying for UPLC, RS, and MCR methods. The obtained results showed good assay results for the examined drugs CFZ and PYD in Kefadim 500 mg &1.0 g IV/IM, as reported in Table 7.

**Table 7:** Assay results for the estimation of CFZ and PYD in their pharmaceutical formulation by the suggested methods

Pharmaceutical formulation	UPLC		UV				Limit %
			RS		MCR		
	CFZ	PYD	CFZ	PYD	CFZ	PYD	
Kefadim 500 mg IV/IM	102.19	0.19	99.86	0.26	100.26	0.22	90–110% for CFZ NMT 0.4% for PYD
	102.07	0.20	102.09	0.25	100.39	0.21	
	103.44	0.20	101.42	0.25	100.64	0.22	
	103.44	0.19	101.61	0.24	100.51	0.22	
	100.91	0.20	103.08	0.26	100.76	0.22	
	100.02	0.19	99.82	0.25	101.01	0.22	
Mean ± RSD	102.01 ± 1.34	0.20 ± 2.80	101.31 ± 1.26	0.25 ± 2.99	100.59 ± 0.27	0.22 ± 1.86	
Kefadim 1.0 g IV/IM	104.85	0.18	99.28	0.27	100.14	0.18	
	102.79	0.18	100.26	0.26	100.51	0.18	
	101.67	0.17	101.58	0.27	100.76	0.18	
	101.85	0.18	103.20	0.26	100.39	0.18	
	103.58	0.17	101.47	0.26	100.89	0.17	
	103.26	0.18	102.15	0.27	99.38	0.18	
Mean ± RSD	103.00 ± 1.15	0.18 ± 2.92	101.32 ± 1.37	0.27 ± 2.06	100.34 ± 0.54	0.18 ± 2.29	

#### Precision

System Precision. The repeatability test was performed by preparing a concentration of 20 µg/mL for each drug in six replicates. The system is precise as RSD for six replicates less than 0.5%, as displayed in Table 3.

Intermediate precision. Laboratory variations, various days, different analysts, and different equipment make ruggedness obvious. Good results were reported in Table 8.

**Table 8:** Ruggedness, Robustness, and stability of analytical solution of the proposed UPLC and UV methods

Parameter	UPLC		UV				Limit %
			RS		MCR		
	CFZ	PYD	CFZ	PYD	CFZ	PYD	
Day to Day	0.82	0.93	0.63	0.81	0.56	0.77	RSD ≤ 2.0%
Analyst to Analyst	0.78	1.20	0.55	0.79	0.71	0.89	
Column to Column	0.54	1.12	–	–	–	–	
Flow rate change (± 0.1 mL/min)	0.95	1.27	–	–	–	–	
pH changes of mobile phase (± 0.2)	0.83	0.88	–	–	–	–	
Wavelength change (± 2.0) nm	0.90	0.96	0.78	0.88	0.83	0.91	
Column temperature change (± 2.0) °C	0.69	0.84	–	–	–	–	
Fresh Sample	0.11	0.20	0.10	0.14	0.13	0.15	
Stored Sample in fridge	0.43	0.56	0.23	0.31	0.25	0.34	
Stored Sample in room temperature	0.74	0.89	0.45	0.52	0.47	0.66	

#### Robustness

The robustness of the analytical proposed methods was executed to confirm that the analytical methods are still competent and unaltered by minor intended alterations in method parameters such as the influence variation in pH, column, temperature, wavelength, and flow rate as recorded in Table 8.

#### Standard solution stability

The standard solution was stocked in different storage conditions as refrigerator and room temperature for 72 h. Then, solutions were analyzed against the freshly prepared solution. The recovered stored standard against the freshly prepared one with good results within the limit of 100% ± 2.0% with RSD < 2.0, as displayed in Table 8.

#### System suitability

BBD was optimized for the chromatographic conditions to select the optimum parameters. The system suitability test was implemented to evaluate the UPLC method through the investigation parameters of the theoretical plates, resolution, tailing factor, and retention time. The acquired results confirm that the UPLC method is suitable, see Table 9.

**Table 9:** System suitability testing specifications of the proposed UPLC method

Item	UPLC		Reference values
	CFZ	PYD	
Tailing factor	0.82	1.1	T ≤ 2
Injection precision	0.22	0.45	RSD ≤ 1%
Number of theoretical plates (N)	6759	4248	N > 2000
Resolution (Rs)	–	2.25	Rs ≥ 1.5

## Conclusion

An eco-friendly, novel, rapid, and highly robust RP-UPLC, RS, and MCR methods were established and validated as per ICH guidelines for concurrently estimation of a binary mixture of CFZ and PYD in their solutions. UPLC highly progresses detection and separation while reducing run time tenfold. BBD and RSM were used to adjust chromatographic conditions with acceptable resolution and the lowest retention time for both drugs using the minimum experimental runs allowed. In addition, Rs and MCR methods were implemented to solve the interference between the binary mixture of CFZ and PYD without prior derivative steps or sophisticated programs. The techniques are also appropriate and valid for use in quality control, lacking HPLC apparatus. Moreover, the application of six sigma was applied to increase product quality and consistency, reduce production costs, and ensure the process is close to the specified center limit. The accelerated stability study was conducted to confirm that active material is not affected by extreme conditions of high temperature and humidity. The proposed methods are valid and can be implemented in the research lab.

## Data Availability

This article has all the data generated or evaluated during this work.

## Abbreviations

RP-UPLC: Reversed phase ultra-performance liquid chromatography
RP-HPLC: Reversed phase high-performance liquid chromatography
UV: Ultraviolet
CFZ: Ceftazidime
PYD: Pyridine
DOE: Design of experiments
BBD: Box-Behnken design
RSM: Response surface methodology
MCR: Mean centering of ratio spectra
RS: Ratio subtraction
Cpk: Process capability index
USP: United States Pharmacopeia
BP: British Pharmacopeia
ICH: International Council for Harmonisation
IV: Intravenously
IM: Intramuscularly
LOD: Limit of detection
LOQ: Limit of quantitation
RSD: Relative standard deviation
